# Supplementary material for: Enterococcus faecium L-15 Cell-Free Extract Improves the Chondrogenic Differentiation of Human Dental Pulp Stem Cells
Source: Int J Mol Sci. 2019 Jan 31;20(3):624. doi: 10.3390/ijms20030624 (PMC6386954; doi:10.3390/ijms20030624)
Supplement: Supplementary file 1 [file ijms-20-00624-s001.pdf]

Supplementary materials

Enterococcus faecium L-15 Cell-Free Extract Improves the Chondrogenic Differentiation of Human Dental Pulp Stem Cells

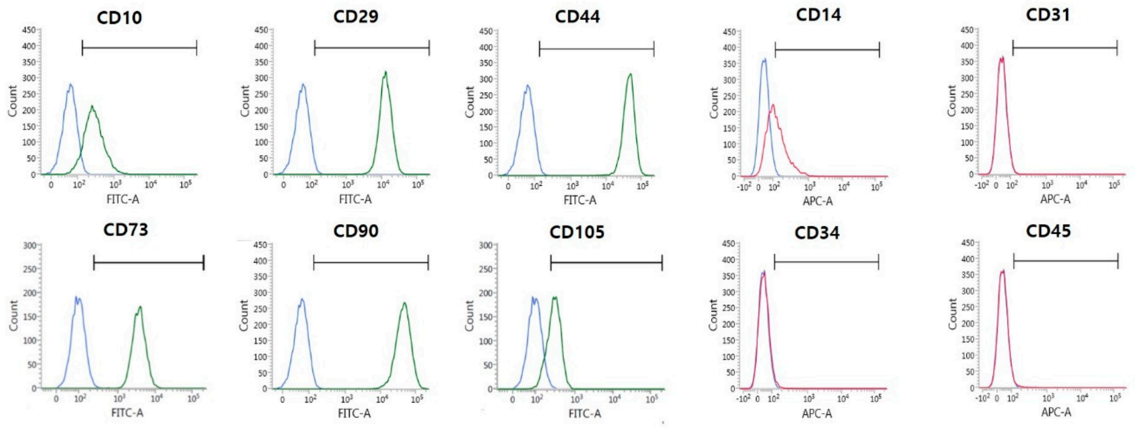

Figure S1. Characterization of hDPSCs at passage 8 by FACS analysis. Mesenchymal stem cell markers (90.05% CD10; 100% CD29; 100% CD44; 100% CD73; 100% CD90; 64.80% CD105) were highly expressed in hDPSCs compared to only a small degree of hematopoietic and endothelial marker expression (49.25% CD14; 1.33% CD31; 3.20% CD34; 2.37% CD45).

Table 1. Surface marker expression of hDPSCs at passage 4 by FACS analysis. Data are presented as average and standard deviation obtained from two donors.

Markers	Average	Standard Deviation	n value (Number of Donors)
CD10	92.48%	3.3	2
CD29	100%	0.0	2
CD44	100%	0.0	2
CD73	100%	0.0	2
CD90	100%	0.0	2
CD105	88.13%	7.77	2
CD14	20.11%	4.22	2
CD31	0.53%	0.23	2
CD34	1.24%	0.91	2
CD45	0.82%	0.37	2

Table 2. Surface marker expression of hDPSCs at passage 8 by FACS analysis. Data are presented as average and standard deviation obtained from two donors.

Markers	Average	Standard Deviation	*n* value (Number of Donors)
CD10	90.05%	3.15	2
CD29	100%	0.0	2
CD44	100%	0.0	2
CD73	100%	0.0	2

CD90	100%	0.0	2
CD105	64.80%	14.58	2
CD14	49.25%	8.365	2
CD31	1.33%	0.795	2
CD34	3.20%	1.78	2
CD45	2.37%	1.885	2

Table 3. Primer sequences used for real-time PCR analysis.

Primer	Forward Primer	Reverse Primer
GAPDH	GCTCTCTGCTCCTCCCTGTTCTAG	TGGTAACCAGGCGTCCGAT
SOX9	AGCGAACGCACATCAAGAC	CTGTAGGCGATCTGTTGGGG
COL2A1	GGCAATAGCAGGTTCACGTACA	CGATAACAGTCTTGCCCCACTT
ACAN	GTGCCTATCAGGACAAGGTCT	GATGCCTTTCACCACGACTTC
COL10A1	CCCTCTTGTTAGTGCCAACC	AGATTCCAGTCCTTGGGTCA
RUNX2	ATACCGAGTGACTTTAGGGATGC	AGTGAGGGTGGAGGGAAGAAG
MMP13	CCAGACTTCACGATGGCATTG	GGCATCTCCTCCATAATTTGGC
